# A quantitative accuracy assessment of the use of a rigid robotic arm in navigated placement of 726 pedicle screws

**DOI:** 10.1186/s12893-022-01838-y

**Published:** 2022-11-10

**Authors:** Carlo Alberto Benech, Rosa Perez, Franco Benech, Torrey Shirk, Brandon S. Bucklen

**Affiliations:** 1 Department of Neurology and Clinical Neurophysiology, Fornaca Clinic, Corso Vittorio Emanuele II, 91 10128 Turin, TO Italy; 2 Musculoskeletal Education and Research Center (MERC), A Division of Globus Medical, Inc, 2560 General Armistead Avenue, Audubon, PA 19403 USA

**Keywords:** Robotic-assisted surgery, Pedicle screw, Computerized tomography navigation, Fluoroscopy

## Abstract

**Background:**

Traditional minimally invasive fluoroscopy-based techniques for pedicle screw placement utilize guidance, which may require fluoroscopic shots. Computerized tomography (CT) navigation results in more accurate screw placement. Robotic surgery seeks to establish access and trajectory with greater accuracy.

**Objective:**

This study evaluated the screw placement accuracy of a robotic platform.

**Methods:**

Demographic data, preoperative/postoperative CT scans, and complication rates of 127 patients who underwent lumbosacral pedicle screw placement with minimally invasive navigated robotic guidance using preoperative CT were analyzed.

**Results:**

On the GRS scale, 97.9% (711/726) of screws were graded A or B, 1.7% (12/726) of screws graded C, 0.4% (3/726) of screws graded D, and 0% graded E. Average offset from preoperative plan to final screw placement was 1.9 ± 1.5 mm from tip, 2.2 ± 1.4 mm from tail and 2.9 ± 2.3° of angulation.

**Conclusions:**

Robotic-assisted surgery utilizing preoperative CT workflow with intraoperative fluoroscopy-based registration improves pedicle screw placement accuracy within a patient’s pedicles.

## Introduction

Minimally invasive (MI) techniques have been integrated into spine surgery in a number of ways, including the placement of percutaneous pedicle screws. As the most common anchor point in posterior spinal fusion procedures, high level of accuracy in screw placement is paramount. Despite relatively low clinically adverse complications associated with malposition, a misplaced screw may still result in neurological/neurovascular injury [1, 2], dural tears [3, 4], sub-optimal biomechanics [5] or other visceral involvement [6]. Furthermore, the number of underreported cases of pedicle malposition are estimated to be as high as 15.7% [3], while the range of pedicle-screw based complications is extremely high (between 1 and 54%), demonstrating that patient conditions and other anatomical factors play a large role in successful screw placement [7]. Several identified patient factors such as challenging deformities, osteoporosis, and tumor have been described [8].

The major modalities for MI spine surgery are fluoroscopic guidance, computed tomography navigation (CTnav), and robot-assisted surgery. The level of evidence in all 3 fields has grown substantially, with several systematic reviews available [9–11]. Fluoroscopy alone, while effective (91.3% accuracy [9]), suffers from obvious radiation and ease-of-use considerations [12, 13], as well as a level of unpredictability based on surgeon experience. Both robotic-guided and CTnav have higher predictability irrespective of experience levels (15.7% [3] vs 7.0% [14] and 5.1% [15] respectively), and most literature supports that robotic-guided surgery has still higher accuracy than fluoroscopic guidance [14]. The use of navigation and robotic assistance results in less radiation exposure than the use of traditional fluoroscopic guidance [16]. Patient factors, such as minimal muscle disruption, quicker recovery times, and less postoperative pain have led to more prevalent uses of this technology [16], while accuracy, safety, and cost appear to be the primary drivers of adoption.

Further data are needed to describe the accuracy and safety of such systems, particularly in a field in which the robotics technologies operate with different hardware, software, and clinical implementation. The aim of present study was to quantitatively assess the accuracy of pedicle screws placed with the guidance and navigation of a robot.

## Materials and methods

This retrospective chart review was exempt from the Italian Ethics Committee. Data were collected from 3 surgeons at a single site. All three surgeons are experienced neurosurgeons who have been doing robotic-assisted surgery since its adoption at their surgical center. Patients who were included in the analysis were between 21 and 85 years of age and required surgery that includes screws to be inserted in thoracic, lumbar spine or sacrum. Demographic data (including age, gender, BMI and diagnosis), operative data (including set up time, screw insertion time, operative time, blood loss, radiation time), preoperative/postoperative computerized tomography (CT) scans, and complication rates of 127 patients treated with lumbosacral pedicle screws through a minimally invasive robotic assisted technique were analyzed. The methods for this publication are similar to those described in a previously published manuscript by the same principal investigators [17].

**Fig. 1:**
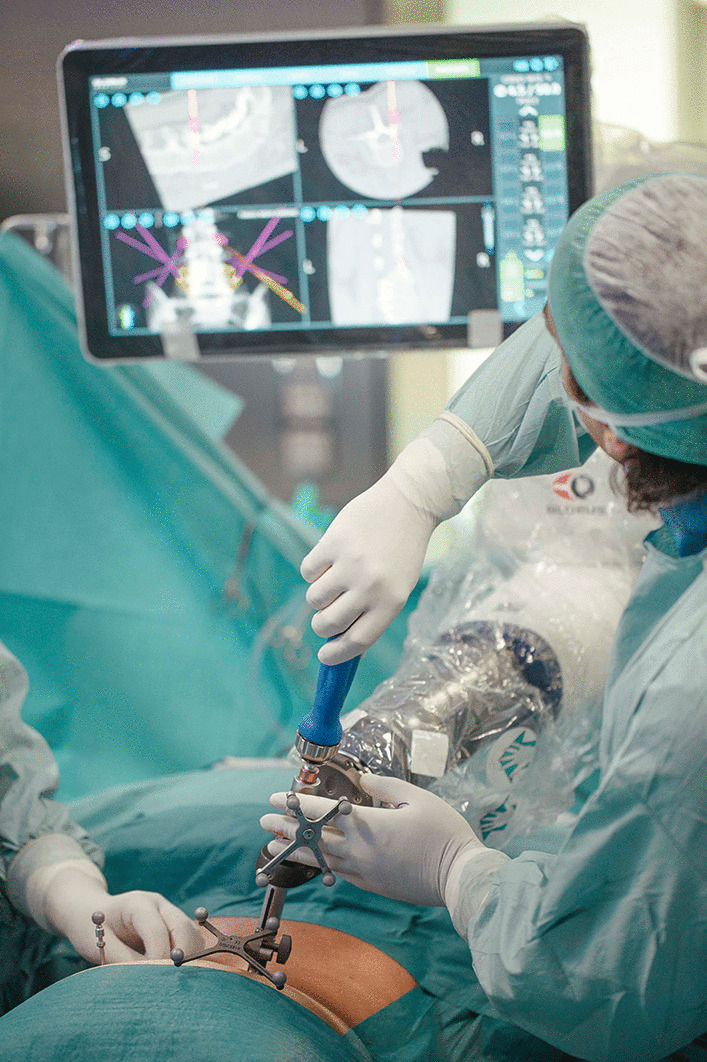
Screw insertion with the robotic positioning system. *Note* This image is the property of the authors, who are the owners of its copyright

The surgical technique employed in this study utilized a robotic positioning system (ExcelsiusGPS®, Globus Medical, Inc., Audubon, PA, USA) (Fig. 1) in which patient radiographs may be uploaded and registered through one of three modalities: preoperative CT, intraoperative CT, or fluoroscopy. For this study, only the preoperative CT workflow was used. In this workflow, a CT scan is taken prior to the surgery, and screw placement planning can be performed. In the operating room (OR), fluoroscopy is used to “merge” the CT and plan to the patient’s positioning on the surgical table. The robotic system uses a dynamic reference base and positioning camera to track the position of instrumentation in real time and 3-dimensional (3D) space, while the rigid robotic arm guides the surgeon to the planned screw trajectory.

### Surgical technique

Once the patient was placed on the table, the dynamic reference base was then placed by making a small incision and anchoring the base to the posterior superior iliac spine. An attachment to the C-arm was used while fluoroscopy shots were taken in view of the robotic system’s positioning camera to merge the patient positioning to the preoperative CT scan. Anterior–posterior and lateral shots were taken to ensure registration of each targeted vertebral level. To confirm successful registration, landmark checks were performed with tracked instruments.

The robotic arm was controlled by the surgeon through a foot pedal, which when pressed caused the arm to move to the preplanned screw trajectory. Through the guide tube attached to the robotic arm, stab incisions were made using a scalpel. Navigated instruments were passed through the guide tube to maintain the planned trajectory, and screws were inserted with these instruments. This process was repeated for all screws. Based on the surgeons’ discretion, 81/127 (63.8%) patients underwent a laminectomy and/or discectomy. Once the screws were placed, rods were placed and held in place with locking caps. Implant position was confirmed with fluoroscopy.

### Accuracy and screw offset

**Fig. 2:**
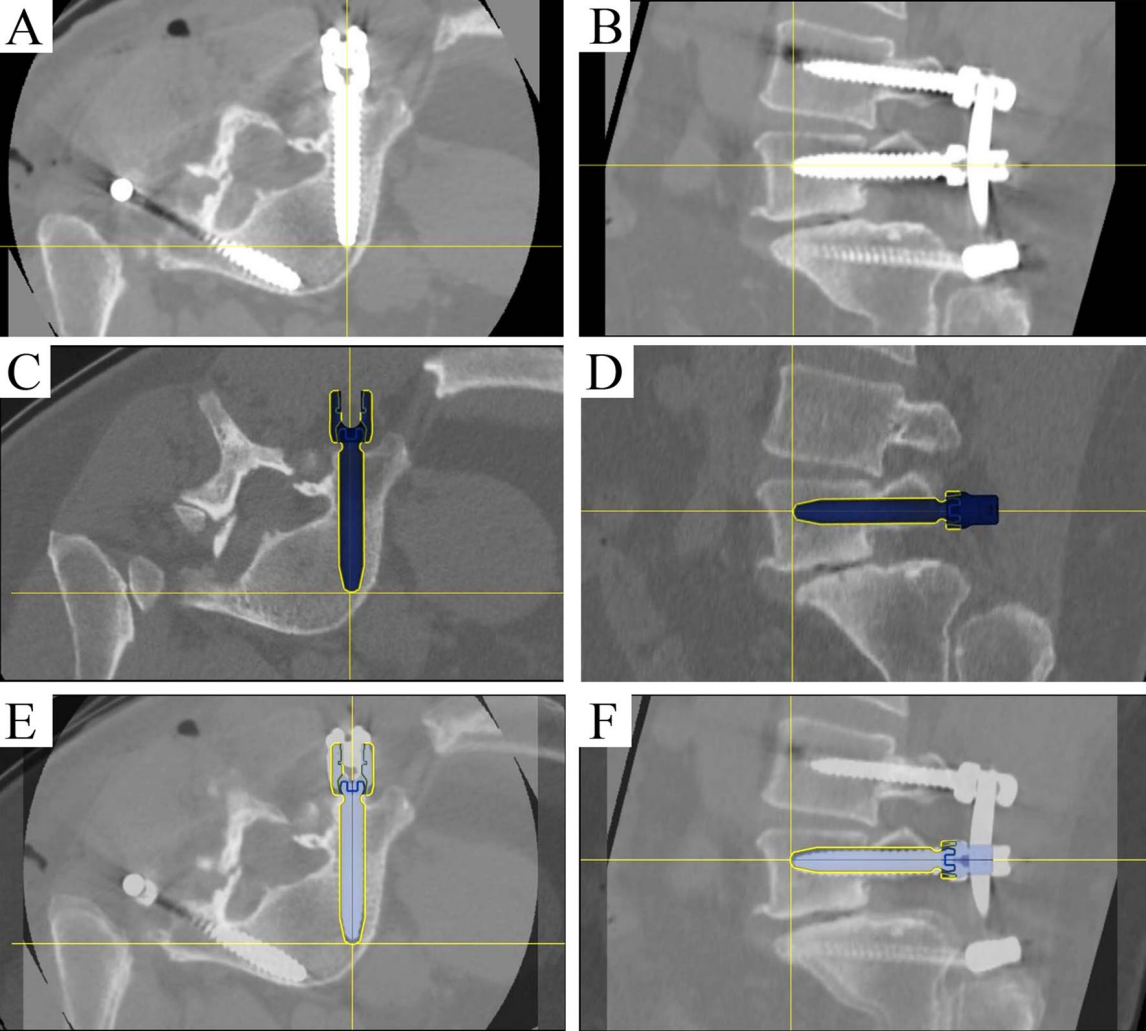
Screw tip, tail, and angle offset assessment. Postoperative CT of L5 screw placement without a medial or lateral breach in **A** axial and **B** sagittal planes. Right L5 screw planning in **C** axial and **D** sagittal planes. Image overlay analysis with preoperative planned trajectory and postoperative screw placement in **E** axial and **F** sagittal planes. The crosshairs indicate screw tip. *Note* This image is the property of the authors, who are the owners of its copyright

Postoperative CT scans were used to perform a Gertzbein and Robbins System (GRS) evaluation of pedicle screw accuracy. In this scale, screws were graded as A (no breach), B (breach of less than 2 mm), C (breach of less than 4 mm), D (breach of less than 6 mm), or E (breach of more than 6 mm). Screws graded to have a less than 2 mm breach (Grade A or B) were considered clinically acceptable, while those screws with a greater than 2 mm breach were considered inaccurate, as in other studies [17–21]. The number of A- and B-graded screws as a percentage of the total screws implanted is presented as an accuracy percentage. Further accuracy analysis utilized postoperative CT scans and the preoperative screw plan trajectories to compare the plan to final placement (Fig. 2). Screw tip, tail, and angulation offsets were measured using software designed for this purpose. Visualizations of planned screws were removed during image overlay to remove potential bias. Screw tip deviation was measured as the difference from planned and final placement at the end, or exit point, of the screw. Screw tail deviation was measured as the difference from the planned and final placement at the head, or entry point, of the screw. These values were 2-dimensional translational measurements, in millimeters, on a screw-centric coordinate system, with the longitudinal axis along the screw being excluded. The 3D angle between the tip and tail vector of the planned trajectory and the vector of the final screw placement was reported as the angular deviation in degrees. Rates of return to OR, screw malposition, and screw repositioning were collected. Patient blood loss was collected for the total surgery time, and during use of the robot system specifically. Blood loss of less than 25 cc was reported as no blood loss. Radiation time was collected and similarly broken out by surgery total and during use of the robotic system. Total operative time and time to place screws was collected.

### Statistical analysis

Statistical analysis was performed using SPSS Statistics Version 25 software (IBM Corp., Armonk, NY, USA). Data were presented as mean ± standard deviation. Pearson correlation coefficients were calculated for the relationship between body mass index (BMI) and screw offset [22]. The level of statistical significance was set to p < 0.05 for all statistical analysis.

## Results

### Patient population

There were 127 patients included in this investigation. The average age was 51.5 ± 11.9 years (range 23–81 years) with 65.4% (83/127) of the patient population being male. The average BMI was 25.5 ± 3.9 kg/m^2^ (range 18.8–40.8 kg/m^2^). Patients were most commonly diagnosed with degenerative disc disease (DDD) (47/127) and degenerative spondylolisthesis (47/127) (Table 1).

**Table 1:** Baseline Characteristics

Parameter	Overall
Number of patients	127
Gender	
Female, n (%)	44 (34.6%)
Male, n (%)	83 (65.4%)
Age, mean ± SD (range)	51.5 ± 11.9 (23–81)
BMI, mean ± SD (range)	25.5 ± 3.9 (18.8–40.8)
Diagnosis, n (%)	
Degenerative Spondylolisthesis	47 (37.0%)
Spondylolisthesis	26 (20.5%)
Degenerative disc disease	47 (37.0%)
Adjacent segment disease	6 (4.7%)
Hardware failure	1 (0.8%)

### Surgical data

There were 732 posterior screws placed with robotic navigation in the 127 patients included in this study. However, 6 screws were not placed with the robot under surgeon discretion and were not included in the analysis, leaving a total of 726 screws analyzed. Average intraoperative set up time was 15.7 ± 8.5 min. Average screw insertion time was 25.4 ± 15.1 min, an average of 4.4 ± 2.2 min per screw. The mean operative time was 121.8 ± 46.8 min, mean blood loss was 44.5 ± 61.6 cc, and mean radiation time was 18.3 ± 13.3 s (Table 2).

**Table 2:** Surgical data

Parameter	Overall
Levels treated, n (%)	
T11	2 (0.3%)
T12	4 (0.5%)
L1	6 (0.8%)
L2	30 (4.1%)
L3	86 (11.7%)
L4	206 (28.1%)
L5	232 (31.7%)
S1	166 (22.7%)
Mean estimated robot blood loss (cc)	5.3 ± 20.7
Mean estimated surgery blood loss (cc)	44.5 ± 61.6
Mean radiation time–robot (s)	9.3 ± 6.1
Mean radiation time–surgery (s)	18.3 ± 13.3
Mean operative time (min)	121.8 ± 46.8
Mean screw insertion time (min)	25.4 ± 15.1

### Tip, tail, and angular offset and screw accuracy

Graded according to the GRS scale, there were 97.9% (711/726) of screws graded A or B, 1.7% (12/726) of screws graded C, 0.4% (3/726) of screws graded D, and 0% graded E. GRS grade by level is presented in Table 3. The average offset from preoperative plan to final screw placement was 1.9 ± 1.5 mm from the tip, 2.2 ± 1.4 mm from the tail and 2.9 ± 2.3° of angulation. Tip, tail and angular offset was not correlated to BMI (tip offset: r = 0.16, p = 0.25; tail offset: r = 0.10, p = 0.47; angular offset: r = 0.03, p = 0.83).

**Table 3:** GRS grade per level

Level treated	Grade A	Grade B	Grade C	Grade D	Grade E
T11	2 (0.3%)	0 (0%)	0 (0%)	0 (0%)	0 (0%)
T12	3 (0.4%)	1 (0.1%)	0 (0%)	0 (0%)	0 (0%)
L1	2 (0.3%)	2 (0.3%)	0 (0%)	0 (0%)	0 (0%)
L2	19 (2.6%)	7 (1.0%)	0 (0%)	0 (0%)	0 (0%)
L3	60 (8.3%)	23 (3.2%)	3 (0.4%)	0 (0%)	0 (0%)
L4	139 (19.1%)	57 (7.9%)	8 (1.1%)	2 (0.3%)	0 (0%)
L5	225 (31.0%)	6 (0.8%)	0 (0%)	1 (0.1%)	0 (0%)
S1	164 (22.6%)	1 (0.1%)	1 (0.1%)	0 (0%)	0 (0%)

### Complications

There was 1 reported complication out of 127 patients. During 1 case, a dural laceration caused the surgeons to open and reposition 4 screws intraoperatively. These 4 screws were excluded from the offset and accuracy analysis. There were no reported returns to the OR. Out of 726 posterior screws placed, 11 (1.5%), including the 4 previously mentioned, were repositioned intraoperatively.

## Discussion

Accurate placement of pedicle screws is essential to avoid complications directly related to screw placement, including dural tears, vascular injury, and compression of the neural elements leading to neurological deficit, among others [23]. Malposition rates of screws implanted without robotic guidance vary widely in the literature from 5.9 to 20.4% [10, 18, 24]. A systematic literature review of robotic screw placement by Joseph et al. [25] found accuracy ranged from 85 to 100% of screws across 25 studies, using a GRS grading of A or B as accurate. In the current study 97.9% of screws were graded either A or B on GRS. This puts the current study in line, but in the 50th percentile of studies compiled in the review done by Joseph et al. [25]. Laudato et al. in a retrospective radiological study from a single academic surgical center, [26] reported a rate of 1.56% of screws determined to be greater than 4 mm breach in a cohort of patients treated with the aid of robot. The current study reports 0.6% (4/726) of screws at this rating. The results of this analysis are similar to other published studies of robotic assisted pedicle screw placement and add to the growing body of literature on the subject.

Placement of the screw within the boundaries of the pedicle is dependent on the accuracy of the screw to the plan developed by the surgeon. Utilizing preoperative CT, the surgeon creates a plan for the placement of each pedicle screw which is registered to the intraoperative fluoroscopy. Close matching to the preoperative plan indicates that the navigation system is accurate in guiding the surgeon during the procedure. Jiang et al. [27] have shown in a similar study, published recently, that accurate screw placement can be achieved with robot with mean deviation of only 1 mm from the planned screw trajectory. Also, in a prospective randomized controlled trial, Han et al. reported the mean deviation of placed screws from planned trajectories was 1.4 ± 0.9 mm for the entry point and 1.6 ± 1.0 mm for the end point. The mean deviation for each screw was 1.5 ± 0.8 mm [28]. In the current study, the average offset from preoperative plan to final screw placement was 1.9 ± 1.5 mm at the tip, 2.2 ± 1.4 mm at the tail and 2.9 ± 2.3° of angulation. Slight deviations from the plan can be expected due to shifting of the patient anatomy intraoperatively, however the values demonstrate that this impact is low. A surprising finding of this study was that neither patient age nor BMI was correlated to screw offset values. Obesity has been shown to be a risk factor of screw misplacement with traditional methods. In one study [8], it was found that obesity significantly increased the odds of misplacement to 3.4. A retrospective analysis of 874 screws [29] determined that BMI was a risk factor for malposition of screws implanted with robotic assistance. The odds ratio for screw misplacement in patients with obesity was found to be 5.4 and statistically significant. Similarly, a comparative study [24] found no difference in the odds ratio for screw misplacement in case of obesity between robotic assisted and freehand placed screws. Absence of a correlation in this study suggests that robotic screw placement in this cohort was not greatly impacted by patient obesity. As obesity may complicate surgical procedures leading to an increased risk of complication [30], this is a finding of note, which requires further investigation. Querying the National Outcomes Database, Onyekwelu et al. found that the obese patients had greater blood loss in surgery, longer surgery times and longer length of stay in hospital postoperatively [31]. Robotic surgery has been shown to reduce operative time [32], potentially mitigating these issues.

The strengths of this study include the large patient size and the addition of surgical/accuracy data to a relatively disparate amount of data that exists for the 3 currently marketed robotic systems, of which the Mazor system (Medtronic, Denver, CO, USA) has been most heavily studied. Limitations of this study are the lack of long-term clinical outcomes to demonstrate the impact of robotic surgery and pedicle screw accuracy on patient health. The current study is from a single location, which impacts the generalizability of the results. Future studies with long-term follow ups from multiple sites under multiple surgical workflows are required to resolve these limitations. Also, future studies can investigate the factors affecting the accuracy of screw placement with the robots.

## Conclusion

Robotic-assisted surgery is highly accurate for the placement of pedicle screws within the patient’s pedicles and according to preoperative planning. In this study, 97.9% of screws were rated either A or B on the Gertzbein–Robbins Scale and tip and tail offset values were an average of 1.9 ± 1.5 mm and 2.2 ± 1.4 mm respectively. Mean intraoperative radiation time was 18.3 ± 13.3 s. Screw placement accuracy is unaffected by patient BMI, a potential benefit of this procedural approach.

## Data Availability

As this is a retrospective review of patient records, raw data were not available and therefore will not be shared in conjunction with this manuscript.

## Abbreviations

CT: Computerized tomography
MI: Minimally invasive
CTnav: Computed tomography navigation
OR: Operating room
3D: 3-Dimensional
GRS: Gertzbein and Robbins System
BMI: Body mass index
DDD: Degenerative disc disease
IRB: Institutional Review Board
IEC: Italian Ethics Committee
